# Health, wellbeing, and the impact of Corona in older adults in Dresden, Germany: the LAB60+ study

**DOI:** 10.1093/eurpub/ckac131.558

**Published:** 2022-10-25

**Authors:** K Romero Starke, J Hegewald, S Schmauder, P Kaboth, L Uhlmann, J Wegge, G Marquardt, A Seidler

**Affiliations:** 1 TU-Dresden, Institute for Occupational Social Medicine, Dresden, Germany; 2 TU-Dresden, Chair of Work and Organisational Psychology, Dresden, Germany; 3 TU-Dresden, Chair of Social and Healthcare Buildings and Design, Dresden, Germany

## Abstract

**Introduction:**

As the proportion of older people increases, it is necessary to evaluate their health and well-being to identify measures to promote healthy ageing. Moreover, the COVID-19 pandemic has impacted older adults’ health- not just through the infection itself, but also due to infection protection ordinances.

**Methods:**

LAB60+ is a population-based cross-sectional study investigating, among other things, the physical and mental health of older adults. Residents of Dresden aged 60 years and older were invited to participate in the first half of 2021. Participants answered questions on their habits, health status and wellbeing, using, among others, the Short Form-8 Health Survey and the WHO wellbeing index.

**Results:**

2399 people participated in the study (40% response). Participants assessed their physical health similar to the German population. The most common chronic conditions were hypertension (54%), chronic pain (32%) and osteoarthritis (31%). 42% of the participants were overweight and 20% were obese. One-fifth did not engage in physical activity: this number increased with age and decreased socioeconomic status. Participants reported lower levels of wellbeing compared to the German population (58 vs. 67 pts.). One-third had higher levels of depressiveness and half reported an increase in their experienced loneliness due to the pandemic. 34% participated in risky alcohol consumption, but it did not markedly change during the pandemic. The greatest negative impact of the pandemic on health behaviors was physical activity: more than one-third exercised less compared to the time before the pandemic.

**Conclusions:**

Physical health was comparable to the German population, while depressivity was higher, perhaps due to the pandemic. Age-appropriate interventions should especially target an increase in physical activity. It is important to take measures to reduce the possible negative effects of the pandemic, such as increased loneliness or reduced physical activity.

**Key messages:**

• This is the first study on health and wellbeing on older adults (60+ years) in the city of Dresden, Germany.

• The COVID-19 pandemic had a high impact on older adults’ physical activity and loneliness.

